# Evaluation of dispensaries’ cannabis flowers for accuracy of labeling of cannabinoids content

**DOI:** 10.1186/s42238-024-00220-4

**Published:** 2024-03-09

**Authors:** Mona M. Geweda, Chandrani G. Majumdar, Malorie N. Moore, Mostafa A. Elhendawy, Mohamed M. Radwan, Suman Chandra, Mahmoud A. ElSohly

**Affiliations:** 1 Department of Pharmaceutics and Drug Delivery, School of Pharmacy, University of Mississippi, University, MS 38677 USA; 2 National Center for Natural Products Research, School of Pharmacy, University of Mississippi, University, MS 38677 USA; 3 Department of Chemistry and Biochemistry, University of Mississippi, University, 38677 USA; 4 Department of Agricultural Biotechnology, Damietta University, Damietta, 34517 Egypt

**Keywords:** Cannabinoids, Δ^9^-THC, Regulations, Label accuracy, Label claim, GC-FID

## Abstract

**Background:**

Cannabis policies have changed drastically over the last few years with many states enacting medical cannabis laws, and some authorizing recreational use; all against federal laws. As a result, cannabis products are marketed in dispensaries in different forms, most abundantly as flowers intended for smoking and sometimes vaping. All samples used in this study were obtained directly from law enforcement. The sample collection process was facilitated and funded by the National Marijuana Initiative (NMI), part of the High-Intensity Drug Trafficking Area (HIDTA) program. This initial report focuses on cannabis flowers. Similar studies with other cannabis products will be the subject of a future report.

**Methods:**

A total of 107 Δ^9^-THC cannabis flower samples were collected by law enforcement from adult commercial use cannabis dispensaries, located in three different states (Colorado, Oregon, and California) and analyzed in this study for cannabinoid concentration. Samples were analyzed by GC-FID following our previously published procedure.

**Discussion:**

The label claims for total Δ^9^-THC content ranged from 12.04 to 58.20% w/w, while GC-FID results showed a concentration ranging from 12.95 to 36.55% w/w. Of the evaluated 107 products, only 32 samples have Δ^9^-THC content within ± 20% of the labeled content. However, the remaining 75 samples were found to be out of the ± 20% acceptance criteria. The degree of agreement for the tested samples using ± 20% tolerance with label claims was only 30%. The results of this study indicate that there is a need for more stringent regulations to ensure that product labeling is accurate, as 70% of the evaluated products did not meet the ± 20% acceptance criteria. This highlights the importance of healthcare professionals and patients being vigilant about the Δ^9^-THC content, as inaccurate labeling of cannabis products could potentially result in adverse health effects. Furthermore, there is a pressing need for more rigorous regulation of commercial cannabis products in the United States.

**Supplementary Information:**

The online version contains supplementary material available at 10.1186/s42238-024-00220-4.

## Introduction

Δ^9^-tetrahydrocannabinol (Δ^9^-THC) is the primary psychoactive component of the cannabis plant. It is responsible for the euphoric effects commonly associated with cannabis use. When consumed, Δ^9^-THC binds to specific cannabinoid receptors in the brain, which can affect mood, memory, and perception. In addition to its psychoactive effects, Δ^9^-THC also has potential therapeutic benefits.

The U.S. Food and Drug Administration (FDA) regulates the use of Δ^9^-THC in certain products, but it does not currently allow the use of THC in foods or dietary supplements (Cascini et al. 2012). This is because THC is classified as a Schedule I controlled substance under the Controlled Substances Act, which means that it has a high potential for abuse and no accepted medical use (Food U 2020).

The FDA has approved some medications that contain synthetic compounds that include Δ^9^-THC, such as dronabinol and nabilone. Dronabinol has been approved for the treatment of nausea and vomiting associated with chemotherapy and for appetite loss associated with the wasting syndrome in patients with HIV/AIDS. However, these medications are only available with a prescription and are strictly regulated by the FDA (Abuhasira et al. 2018).

While cannabis is illegal at the federal level, many states have legalized the use of cannabis for medical or recreational purposes. In February 2022, National Conference of State Legislatures declared that 37 states, three territories, and the District of Columbia allow the medical use of cannabis products (National Conference of State Legislatures 2023). The use of medical cannabis has been reported to be associated with improvements in symptoms related to a variety of medical conditions, including chronic pain, nausea and vomiting associated with chemotherapy, and spasticity associated with multiple sclerosis (Spindle et al. 2019; Kogan and Mechoulam 2007). However, the use of cannabis, especially for recreational purposes, can be associated with risks, including addiction, impaired driving, and potential adverse effects on mental health (Hall 2015).

The shift in cannabis policies has led to a surge of cannabis products with diverse concentrations of cannabinoids that are flooding the markets in the United States. However, one of the significant challenges associated with these products is accurate labeling. Ensuring that these products are appropriately labeled is crucial in mitigating or preventing adverse consequences that can arise when the information is incomplete, unreliable, or insufficiently informative. In particular, accurate labeling can help reduce risks such as excessive consumption, improper dosing, and acute adverse events. Therefore, it is essential to determine the accuracy and reliability of the labeling in order to promote informed decision-making and responsible use of cannabis products (Vandrey et al. 2015; Hammond 2021).

While cannabis-based products contain a range of cannabinoids that include ∆^9^ – THC, CBD, CBG, CBC, THCV and CBN, most testing of marketed products is directed toward CBD and THC levels. ∆^9^-THC, being a psychoactive compound falls under schedule-I drug category, is supposed to be below 0.3% in the products reported as hemp. Therefore, testing of hemp products includes the ∆^9^-THC level in addition to CBD.

Research has indicated that inaccurate labeling of CBD products is a widespread problem not limited to the United States. For example, a study of 84 CBD products found that only 31% of the products were accurately labeled within 10% of the advertised CBD content (Bonn-Miller et al. 2017). Similarly, a study conducted in Mississippi found that only 2 out of 20 CBD products were within the 10% accuracy range of the advertised CBD content (Gurley et al. 2020). In the Netherlands, a study of 16 CBD oil products revealed that only 5 products contained CBD within 10% of the labeled amount (Hazekamp 2018). Additionally, a study in Italy involving 14 CBD oil products showed that only 5 of the products contained CBD consistent with the labeled content (within 10%) (Pavlovic et al. 2018). Similarly, a UK-based study reported that only 11 out of 29 CBD oil products tested contained CBD within 10% of the advertised amount (Liebling et al. 2022). These findings highlight the pervasive issue of inaccurate labeling of CBD products, which can have significant implications for consumer safety and highlights the need for greater regulatory oversight and standards in the industry.

## Materials and methods

### Sample selection

A total of 107 dried cannabis flower samples were obtained from state Law enforcement personnel, the National Cannabis Initiative (NMI). These samples were randomly selected by the state Law enforcement team from each state. Samples were received from three states: Colorado (23 samples), Oregon (16 samples), and California (68 samples, with 47 from San Diego and 21 from the Central Valley region). The plant samples were comprised of different brands. Each product was randomly assigned a study identifier to blind researchers to product identification. Upon receipt, product packaging and seals were inspected to ensure product integrity. The lot numbers were recorded, and the claimed amounts on the products were acquired from a label on the product. The products were stored according to packaging instructions or at room temperature in a dry space if instructions were not provided. All products were tested immediately after opening.

For product label accuracy, an acceptance criteria of ± 20% was applied (Sarma et al. 2020). If Δ^9^-THC concentration is more than 120% of the labeled value, the product was under-claimed, but if the value is less than 80% of the labeled value the product was over-claimed. Products within ± 20% (i.e., 80–120% of labeled value) are categorized as accurately labeled**.**

### Cannabinoid standards and calibration curves

Standard solutions of seven pure cannabinoids (THCV, CBD, CBC, Δ^8^-THC, Δ^9^-THC, CBG, and CBN) were isolated in our laboratory with a purity greater than 95% (Ahmed et al. 2008; Husni et al. 2014). The analysis was carried out following the GC-FID method previously described (ElSohly et al. 2016). The cannabinoids standards were used to prepare the calibration curves used for the quantification of the individual cannabinoids.

### Sample preparation for GC-FID analysis

Two samples (100 mg each) from each product were analyzed and the average content was used for the label accuracy (the results of the two analysis cannot differ by more than ± 20%; otherwise analysis is repeated again in duplicate). Each sample was extracted with 3 ml of internal standard (I.S.) solution, which contained 1 mg/ml of 4-Androstene-3,17-dione in CH_3_OH/CHCl_3_ (9:1), for one hour. The resulting mixture was then filtered to create a working solution for GC analysis where 1 μL of the extracted material is injected on the GC/FID. Analysis was carried out using a Varian 3380 gas chromatography system, which was equipped with a Varian CP-8400 automatic liquid sampler, dual capillary injectors, and dual flame ionization detectors (GC/FID). The instrument parameters were as follows: air at 30 psi (300 mL/min), hydrogen at 30 psi (30 mL/min), the carrier gas is helium with column head pressure of 14 psi (1.0 mL/min), the split ratio at 15:1, septum purge flow rate at 3 mL/min, makeup gas (helium) pressure at 20 psi (30 mL/min), injector temperature at 240 °C, detector temperature at 270 °C, oven temperature was programmed starting at 170 °C (hold for 1 min) and ramping up to 250 °C at 10 °C/min (hold for 3 min), with a total run time of 12 min. The GC column used was J&W DB-1 Agilent with dimensions of 15 m (length) × 0.25 mm (diameter) and a 0.25 μm wall thickness (ElSohly et al. 2016).

### Method validation

The method used for this analysis was validated as per the ICH method validation guidelines for the following parameters; specificity, linearity and range, sensitivity, and precision (ICH Guideline 1996).

### Specificity

**Fig. 1:**
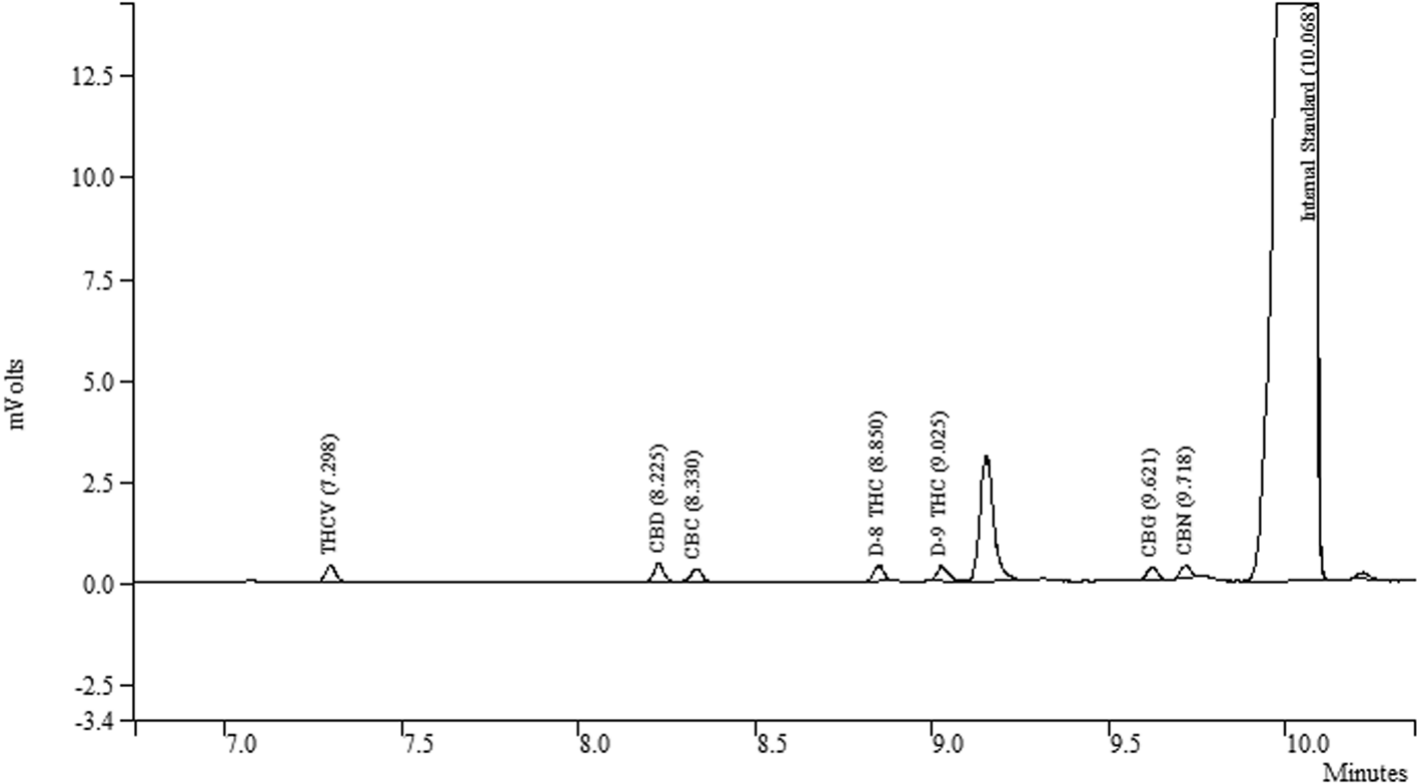
Chromatogram of a sample prepared at the lower limit of quantification (LLOQ) of all of the analyzed cannabinoids

**Fig. 2:**
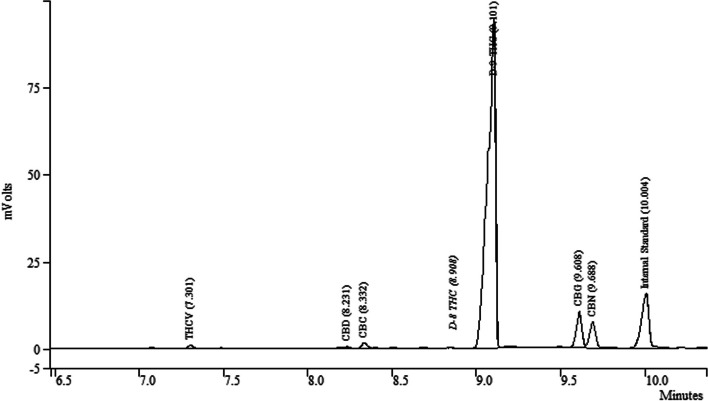
Chromatogram of a representative commercial sample

The method specificity was determined as no interfering peaks were found at the retention time of any of the target cannabinoids. Moreover, the qualitative and quantitative analysis of samples was performed by comparing their retention times with the reference standard of each analyzed cannabinoid. The method provided baseline separation for all of the seven cannabinoids. The chromatogram of a sample prepared at the lower limit of quantification (LLOQ) of all cannabinoids is shown in Fig. 1. Furthermore, a chromatogram of one of the commercial samples here is shown in Fig. 2.

### Linearity and range

**Fig. 3:**
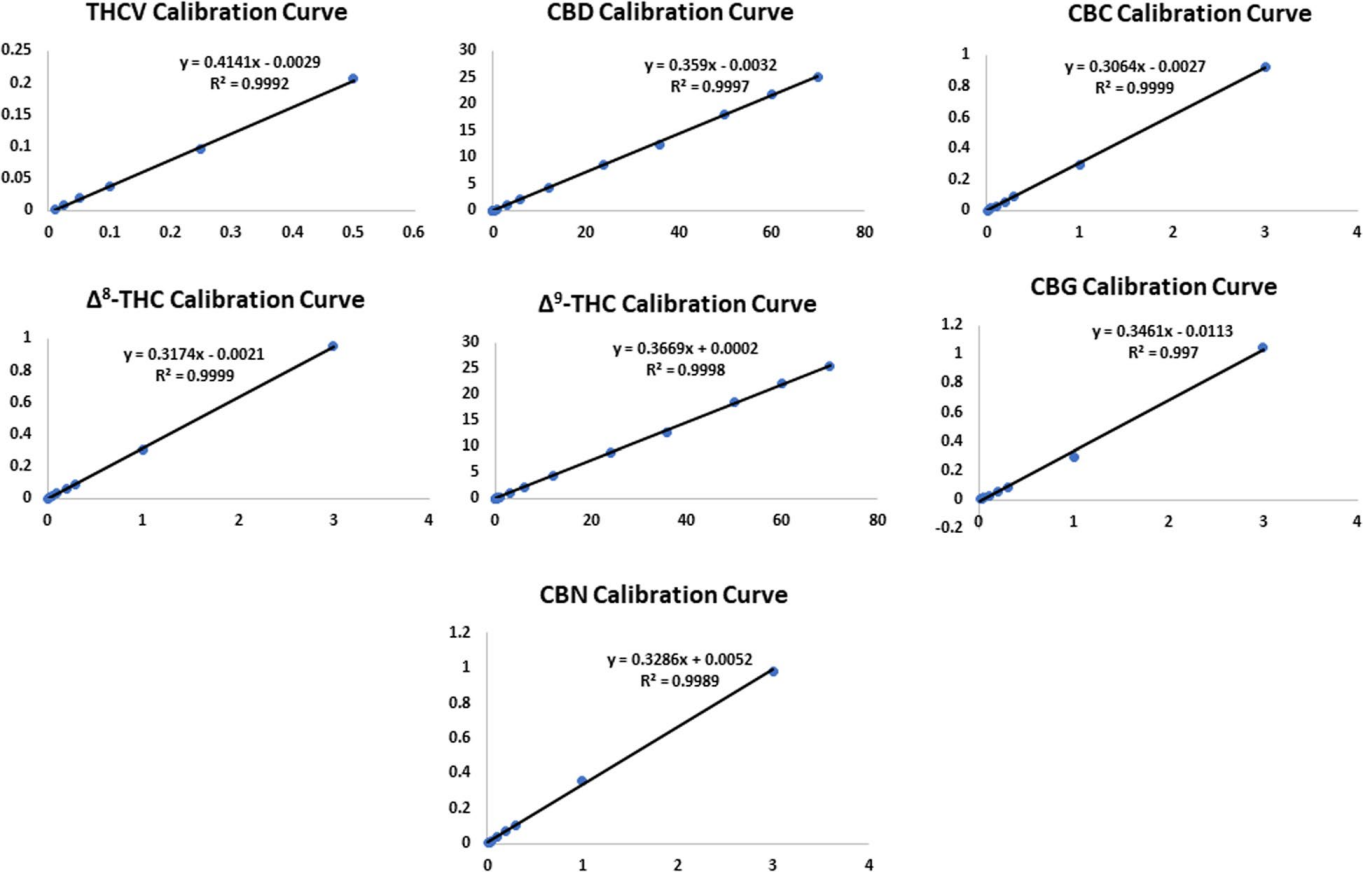
Calibration curves of the analyzed cannabinoids

Linearity and range samples were prepared by spiking placebo plant material with different amounts of the target cannabinoids. For THC and CBD, the calibration range was 0.01–70% w/w, while for THCV was 0.01–0.5% w/w. On the other hand, Δ^8^-THC, CBC, CBG, and CBN calibration range was 0.01–3% w/w. The selection of the calibration range was based on the expected prevalence of the tested cannabinoids in the plant material. Calibration curves were constructed by plotting the concentration of each cannabinoid against the peak area ratio (peak area of each cannabinoid/peak area of the I.S.). The correlation coefficient of the regression line(R^2^) for each cannabinoid ranged from 0.997–0.9999 (Fig. 3). The detailed results of regression parameters are shown in Table 1.

**Table 1:** Regression equations, R^2^, Calibration range, LOD, LOQ and retention time (min.) of the tested cannabinoids

Compound	Regression equation	*R*^2^	Calibration range (% w/w)	LOD (% w/w)	LOQ (% w/w)	Retention time (min.)
**THCV**	y = 0.4141x–0.0029	0.9992	0.01–0.5	0.005	0.01	7.30
**CBD**	y = 0.359x–0.0032	0.9997	0.01–70	0.005	0.01	8.23
**CBC**	y = 0.3064x–0.0027	0.9999	0.01–3	0.005	0.01	8.33
**Δ**^**8**^**-THC**	y = 0.3174x–0.0021	0.9999	0.01–3	0.005	0.01	8.85
**Δ**^**9**^**-THC**	y = 0.3669x + 0.0002	0.9998	0.01–70	0.005	0.01	9.03
**CBG**	y = 0.3461x–0.0113	0.997	0.01–3	0.005	0.01	9.62
**CBN**	y = 0.3286x + 0.0052	0.9989	0.01–3	0.005	0.01	9.72
**I.S**	-	-	-	-	-	10.07

### Method sensitivity (limit of detection (LOD) and limit of quantification (LOQ))

Method sensitivity was assessed by LOD and LOQ. The LOD and LOQ concentrations were 0.005 and 0.01%w/w for all the tested cannabinoids, respectively. The LOD and LOQ were evaluated based on signal-to-noise ratios greater than 3 and 10, respectively.

### Precision

Method precision was evaluated using six different sample preparations of cannabis plant material (High THC chemovar). The intermediate precision was evaluated by data generated on three different days. The %RSD was less than 8% for all the tested cannabinoids indicating the method is precise (Table 2).

**Table 2:** Precision (intra-day and inter-day) results

	Day 1 (*n* = 6)			Day 2 (*n* = 6)			Day 3 (*n* = 6)			Between Days (*n* = 18)		
Compound	**Mean**	**SD**	**%RSD**	**Mean**	**SD**	**%RSD**	**Mean**	**SD**	**%RSD**	**Mean**	**SD**	**%RSD**
**THCV**	0.031	0.002	7.83%	0.032	0.000	1.09%	0.032	0.000	0.97%	0.032	0.0008	2.63%
**CBD**	4.365	0.273	6.26%	4.694	0.098	2.08%	4.804	0.085	1.76%	4.621	0.2280	4.93%
**CBC**	0.269	0.016	5.80%	0.294	0.013	4.31%	0.299	0.009	2.87%	0.288	0.0160	5.56%
**D8-THC**	0.017	0.0004	2.64%	0.016	0.0004	2.20%	0.016	0.0002	0.94%	0.017	0.0003	1.85%
**D9-THC**	2.930	0.054	1.83%	3.012	0.037	1.24%	3.024	0.018	0.59%	2.989	0.0512	1.71%
**CBG**	0.269	0.007	2.71%	0.281	0.004	1.31%	0.274	0.003	1.19%	0.274	0.0062	2.27%
**CBN**	0.215	0.004	1.73%	0.210	0.003	1.23%	0.212	0.003	1.40%	0.212	0.0025	1.20%

### Statistical analysis

Cannabinoid profiling was performed for each product sample. Descriptive statistics were used to report the mean (SD) claimed amounts and observed amounts of THC in the products from each regional/state area.

Classification was based on a ± 20% acceptance range, with products classified as under-claimed, over-claimed, or accurately labelled. All calculations were completed using Microsoft Excel version 16.66 and JASP version 0.16.4.

If the claimed value is higher than the observed value, the % variance is calculated as follows:

$$\left\{\mathrm{Claimed}\;\mathrm\Delta^9\mathrm{THC}\;\mathrm{content}\;(\%)\;-\;\mathrm{Observed}\;\mathrm\Delta^9\mathrm{THC}\;\mathrm{content}\;(\%)\right\}\times100/\mathrm{Observed}\;\mathrm{value}$$

If the observed value is higher than the claimed value, the % variance is calculated as follows:

$$\left\{\mathrm{Observed}\;\mathrm\Delta^9\mathrm{THC}\;\mathrm{content}\;(\%)\;-\;\mathrm{Claimed}\;\mathrm\Delta^9\mathrm{THC}\;\mathrm{content}\;(\%)\right\}\times100/\mathrm{Observed}\;\mathrm{value}$$

## Results and discussion

A total of 107 dried cannabis flower marketed products were analyzed for their content of Δ^9^-THC along with six other cannabinoids (THCV, CBD, CBC, Δ^8^-THC, CBG, and CBN). The Δ^9^-THC content as % w/w was determined using GC-FID and the results were compared to label claims on the packages. The samples were collected from three states, namely Colorado, Oregon and California (Two sites: San Diego and Central Valley). Table 3 shows the Descriptive statistics of Δ^9^-THC concentration in dispensary cannabis flowers (observed vs claimed). The data show that for all sites of samples collection, the average THC content claimed by the manufacturers was inflated by up to 30% from the observed mean values. Details of variance of each individual sample are shown in Tables 4, 5, 6 and 7. Other cannabinoids analysis (THCV, CBC, CBG, CBN and Δ^8^-THC) of samples collected from Colorado; Oregon; San Diego, CA and Central Valley, CA is shown in the Table 1S, Table 2S, Table 3S and Table 4S, respectively (pl. see Supplementary information). While only THC levels are shown in Table 4, CBD values are not reported, first because the labels did not include CBD levels and second, our analysis showed CBD levels of all samples were < 0.1%. However, respective CBD labels are incorporated to Table 1S, 2S, 3S, and 4S.

**Table 3:** Descriptive statistics of Δ^9^-THC concentration in dispensary cannabis flowers (observed vs claimed)

Parameters	**States**							
	**Colorado**		**Oregon**		**California**			
					**San Diego**		**Central Valley**	
	**Claimed Δ**^**9**^**-THC**	**Observed Δ**^**9**^**-THC**	**Claimed Δ**^**9**^**-THC**	**Observed Δ**^**9**^**-THC**	**Claimed Δ**^**9**^**-THC**	**Observed Δ**^**9**^**-THC**	**Claimed Δ**^**9**^**-THC**	**Observed Δ**^**9**^**-THC**
# of Samples Collected	23	23	16	16	47	47	21	21
Mean	28.56	21.22	29.52	24.55	28.52	20.90	32.97	23.51
Std. Deviation	8.30	3.97	7.43	6.88	5.72	3.84	8.47	4.40
Minimum	17.60	13.14	12.04	15.37	15.84	12.95	24.56	17.32
Maximum	53.44	30.32	37.22	36.55	38.00	28.96	58.20	34.66

**Table 4:** Claimed vs observed ∆^9^- THC content (%) of the 23 Colorado samples

Sample Code	Claimed ∆^9^- THC	Observed ∆^9^- THC	% Variance
CO1	19.5	19.84	2%
CO2	24.6	24.62	0%
CO3	17.6	19.01	7%
CO4	20.2	18.29	10%
CO5	21.15	17.51	21%
CO6	22.89	23.41	2%
CO7	26.8	20.88	28%
CO8	29.86	25.57	17%
CO9	27.99	15.58	80%
CO10	33.1	26.63	24%
CO11	24.4	19.70	24%
CO12	27.3	17.68	54%
CO13	30	17.93	67%
CO14	30	24.32	23%
CO15	31.96	30.32	5%
CO16	37.84	22.40	69%
CO17	53.44	26.65	101%
CO18	21.05	19.20	10%
CO19	39.3	21.00	87%
CO20	36.31	20.91	74%
CO21	27.24	20.81	31%
CO22	18.9	13.14	44%
CO23	35.42	22.69	56%

There were 23 samples from the state of Colorado. Table 4 shows the observed vs the claimed Δ^9^-THC values for each product. The claimed Δ^9^-THC content in these products ranged from 17.6% to 53.44% while the observed values ranged from 13.14% to 30.32%. Among the 23 Colorado samples only 8 samples (35% of the samples) were within ± 20% difference between the claimed and observed values, while 65% of the samples (15 out of 23 samples) were outside ± 20% acceptance range, and the overclaimed THC values were as much as twice the actual values.

Table 5 shows a comparison between the observed vs the claimed of Δ^9^-THC content of the 16 cannabis flower products obtained from Oregon. The claimed Δ^9^-THC values in these products varied from 12.04% to 37.22%, whereas the observed values ranged from 15.37% to 36.55%. Of the 16 samples, 7 samples (44% of the samples) were within the ± 20% acceptance range. Nine samples (56% of the samples) were outside ± 20% acceptance range.

**Table 5:** Claimed vs observed ∆^9^- THC content (%) of the 16 Oregon samples

Sample Code	Claimed ∆^9^- THC	Observed∆^9^- THC	% Variance
OR 1	30.40	17.91	70%
OR 2	32.44	17.24	88%
OR 3	31.38	33.99	8%
OR 4	31.9	36.55	13%
OR 5	37.22	27.01	38%
OR 6	36.22	23.47	54%
OR 7	35.9	29.60	21%
OR 8	30.29	21.91	38%
OR 9	31.31	31.64	1%
OR 10	35.83	31.98	12%
OR 11	18.78	17.27	9%
OR 12	25.14	18.97	33%
OR 13	12.04	15.37	22%
OR 14	36.05	26.99	34%
OR 15	29.67	25.94	14%
OR 16	17.72	16.91	5%

Finally, there was 68 samples received from two different sites in the state of California (San Diego, 47 samples and Central Valley, 21 samples) as shown in Tables 6 and 7. Table 6 presents the observed vs the claimed Δ^9^-THC content (%) of each sample obtained from San Diego, California. The claimed Δ^9^-THC values of these products had a minimum value of 15.84% and a maximum value of 38%, while the observed values ranged from 12.95% to 28.96%. Among the 47 samples, only 14 samples (30% of the samples) were found to fall within the ± 20% difference between the claimed and observed values, whereas 33 samples (70% of the samples) were outside the ± 20% acceptance range. Thus, from the data shown in Table 6, it is evident that the overstated THC values claimed in the San Diego samples ranged from 24% (sample # 9) to 160% (sample #37).

**Table 6:** Claimed vs observed ∆^9^- THC content (%) of the 47 San Diego, California samples

Sample Code	Claimed ∆9- THC	Observed ∆9- THC	% Variance
SD Cal 1	20.7	20.77	0%
SD Cal 2	23.26	21.57	8%
SD Cal 3	23.17	17.85	30%
SD Cal 4	32.82	21.93	50%
SD Cal 5	15.84	15.06	5%
SD Cal 6	28.87	23.95	21%
SD Cal 7	27.07	24.41	11%
SD Cal 8	30.76	26.27	17%
SD Cal 9	25.47	20.57	24%
SD Cal 10	25.52	20.04	27%
SD Cal 11	25.12	18.49	36%
SD Cal 12	27.56	23.42	18%
SD Cal 13	23.76	20.39	17%
SD Cal 14	26.89	26.60	1%
SD Cal 15	31.23	24.71	26%
SD Cal 16	34.36	27.47	25%
SD Cal 17	31.1	19.89	56%
SD Cal 18	21.32	16.28	31%
SD Cal 19	30.23	23.05	31%
SD Cal 20	26.7	24.41	9%
SD Cal 21	34.79	26.54	31%
SD Cal 22	23.08	19.98	16%
SD Cal 23	32.72	24.56	33%
SD Cal 24	25.2	19.44	30%
SD Cal 25	22.97	19.62	17%
SD Cal 26	17.84	16.29	10%
SD Cal 27	38	22.07	72%
SD Cal 28	37	23.60	57%
SD Cal 29	31.34	17.80	76%
SD Cal 30	27.39	18.05	52%
SD Cal 31	36.15	28.96	25%
SD Cal 32	32.6	20.92	56%
SD Cal 33	23.72	21.93	8%
SD Cal 34	24.58	19.76	24%
SD Cal 35	32	23.10	39%
SD Cal 36	28.57	26.96	6%
SD Cal 37	35.64	13.70	160%
SD Cal 38	32.42	20.31	60%
SD Cal 39	20.2	14.99	35%
SD Cal 40	26.69	12.95	106%
SD Cal 41	34.51	21.34	62%
SD Cal 42	36.72	19.91	84%
SD Cal 43	37.06	20.67	79%
SD Cal 44	35.3	22.42	57%
SD Cal 45	20.08	13.68	47%
SD Cal 46	26	15.63	66%
SD Cal 47	36.27	19.74	84%

**Table 7:** Claimed vs observed ∆^9^- THC content (%) of the 21 Central Valley, California samples

Sample Code	Claimed ∆^9^- THC	Observed ∆^9^- THC	% Variance
CV Cal 1	33.70	26.78	26%
CV Cal 2	28.57	25.21	13%
CV Cal 3	37.9	27.29	39%
CV Cal 4	29	19.6	48%
CV Cal 5	25.05	19.78	27%
CV Cal 6	25.10	18.65	35%
CV Cal 7	35.27	26.24	34%
CV Cal 8	26.37	18.63	42%
CV Cal 9	58.20	28.93	101%
CV Cal 10	33.63	23.93	41%
CV Cal 11	33.41	22.64	48%
CV Cal 12	40	26.52	51%
CV Cal 13	29.48	19.98	48%
CV Cal 14	25.98	17.32	50%
CV Cal 15	30.32	20.83	46%
CV Cal 16	35.14	28.13	25%
CV Cal 17	26.25	19.91	32%
CV Cal 18	50.1	26.05	92%
CV Cal 19	28.22	20.29	39%
CV Cal 20	36.19	34.66	4%
CV Cal 21	24.56	22.23	10%

As for the 21 samples received from Central Valley California, the observed vs the claimed Δ^9^-THC content of each sample is displayed in Table 7. The claimed Δ^9^-THC content in these products ranged from 24.56% to 58.20%, while the observed values varied from 17.32% to 34.66%. Only 3 samples out of 21 (14% of the samples) were within the ± 20% difference, while 18 samples (86% of the samples) were outside the ± 20% acceptance range, (all had inflated values) making the samples from Central Valley California to have the highest overstated level of Δ^9^-THC on their labels among all samples tested.

While the lower observed THC content might be because of the degradation of THC during storage (the age of samples is not known), this cannot be the main reason. Examination of the CBN concentration in all samples (see Supporting data) showed that only few samples (eight) contain CBN at levels of > 1%. Indication that degradation of THC overtime cannot explain the high levels of overstating the THC content.

## Conclusions

The results of this study indicate that there is a need for more stringent regulations to ensure that product labeling is accurate, as over 70% of the evaluated products did not meet the ± 20% acceptance criteria. It is noted that out of all samples that were found to be mislabeled (outside of ± 20% difference between claimed values and analytical values), only one sample (#13, Table 5) was under labelled, while all others were over labelled. That might be because of the higher economic gain by stating a higher Δ^9^-THC content. Another possibly reason for the overstated values could be the loss of THC content as a result of storage under unfavorable conditions. That possibly was not considered because all products were analyzed prior to the stated expiration date. Analytically speaking, the method was properly validated and many of the products (approx. 30%) did fall within the acceptable range, therefore there are no limitations caused by the method.

Furthermore, several research groups, as well as the FDA, have conducted investigations into the accuracy of CBD products labeling, and their findings consistently highlight concerns about the accuracy of the information provided. For example, between 2015 and 2016, the FDA issued warning letters to 14 businesses regarding their products containing less CBD than advertised, with some instances of negligible or less than 1% of the claimed content. Additionally, seven products were found to contain THC levels exceeding the statutory limit of 0.3% (w/w), yet no information was provided on the labels about these levels. These findings underscore the need for greater regulation and oversight in the CBD industry to ensure transparency and accuracy in labelling, which can help protect consumers from potentially harmful effects (FDA 2021). In the meantime, our limited amount of data highlights the importance of healthcare professionals and patients being vigilant about the Δ^9^-THC content, as inaccurate labeling of cannabis products could potentially result in adverse health effects.

## Supplementary Information

**Supplementary Material 1.**

**Supplementary Material 2.**

**Supplementary Material 3.**

**Supplementary Material 4.**

## Data Availability

All data generated or analyzed during this study are included in this published article.

## Abbreviations

CBC: Cannabichromene
CBD: Cannabidiol
CBG: Cannabigerol
CBN: Cannabinol
Δ^8^-THC: Δ^8^-Tetrahydrocannabinol
Δ^9^-THC: Δ^9^-Tetrahydrocannabinol
FDA: Food and Drug Administration
GC-FID: Gas Chromatography with Flame Ionization Detector
NMI: National Marijuana Initiative
THCV: Tetrahydrocannabivarin
