# Supplementary material for: Evaluation of dispensaries’ cannabis flowers for accuracy of labeling of cannabinoids content
Source: J Cannabis Res. 2024 Mar 9;6:11. doi: 10.1186/s42238-024-00220-4 (PMC10924369; doi:10.1186/s42238-024-00220-4)
Supplement: Supplementary file 1 — Supplementary Material 1. [file 42238_2024_220_MOESM1_ESM.pdf]

Table 1S. Observed Cannabinoids profile (%) of CBD, THCV, CBC, Δ^8-THC, CBG, and CBN in Colorado.

Sample Code	CBD	THCV	CBC	Δ^8- THC	CBG	CBN
CO1	0.04	0.11	0.33	0.48	0.77	0.13
CO2	0.10	0.11	0.28	0.51	0.42	0.09
CO3	0.04	0.08	0.22	0.01	0.51	0.12
CO4	0.04	0.09	0.30	<0.01*	0.46	0.12
CO5	0.04	0.08	0.33	0.01	0.78	0.21
CO6	0.04	0.08	0.65	0.03	0.72	0.12
CO7	0.04	0.13	0.47	0.11	1.13	0.25
CO8	0.05	0.15	0.27	0.71	0.92	0.10
CO9	0.03	0.07	0.22	0.05	0.20	0.09
CO10	0.05	0.11	0.26	0.32	0.04	1.18
CO11	0.04	0.18	0.18	0.09	0.61	0.11
CO12	0.03	0.10	0.21	0.28	1.07	0.09
CO13	0.04	0.07	0.34	0.01	0.78	0.18
CO14	0.05	0.11	0.26	0.13	0.76	0.13
CO15	0.05	0.16	0.41	0.28	0.48	0.21
CO16	0.05	0.06	0.32	0.01	0.38	0.11
CO17	0.05	0.21	0.29	0.49	0.39	0.08
CO18	0.04	0.20	0.30	0.47	0.67	0.15
CO19	0.04	0.21	0.30	0.01	0.62	0.12
CO20	0.04	0.07	0.25	0.21	0.55	0.41
CO21	0.07	0.09	0.21	0.11	0.52	0.62
CO22	0.04	0.06	0.16	0.33	0.13	1.53
CO23	0.07	0.07	0.32	0.10	0.78	0.35
