# Supplementary material for: Evaluation of dispensaries’ cannabis flowers for accuracy of labeling of cannabinoids content
Source: J Cannabis Res. 2024 Mar 9;6:11. doi: 10.1186/s42238-024-00220-4 (PMC10924369; doi:10.1186/s42238-024-00220-4)
Supplement: Supplementary file 2 — Supplementary Material 2. [file 42238_2024_220_MOESM2_ESM.pdf]

Table 2S. Observed Cannabinoids profile (%) of CBD, THCV, CBC, Δ^8- THC, CBG, and CBN in Oregon.

Sample Code	CBD	THCV	CBC	Δ^8- THC	CBG	CBN
OR 1	0.04	0.09	0.16	0.10	0.42	1.95
OR 2	0.08	0.11	0.21	0.18	0.60	0.08
OR 3	0.07	0.17	0.30	0.13	0.50	0.11
OR 4	0.06	0.16	0.99	0.48	2.00	0.43
OR 5	0.06	0.08	0.26	0.12	0.63	0.30
OR 6	0.06	0.10	0.24	0.26	1.27	0.27
OR 7	0.08	0.13	0.47	0.34	0.96	0.30
OR 8	0.04	0.10	0.35	0.32	0.30	1.18
OR 9	0.07	0.05	0.29	0.31	0.84	0.12
OR 10	0.06	0.15	0.44	0.34	1.20	0.28
OR 11	0.03	0.11	0.47	0.37	2.39	0.28
OR 12	0.05	0.09	0.32	<0.01*	0.87	0.15
OR 13	0.03	0.14	0.17	0.14	0.29	0.18
OR 14	0.08	0.18	0.31	<0.01*	0.66	0.19
OR 15	0.07	0.17	0.24	0.22	1.30	0.12
OR 16	0.04	0.46	0.35	<0.01*	1.28	0.10
