# Supplementary material for: Evaluation of dispensaries’ cannabis flowers for accuracy of labeling of cannabinoids content
Source: J Cannabis Res. 2024 Mar 9;6:11. doi: 10.1186/s42238-024-00220-4 (PMC10924369; doi:10.1186/s42238-024-00220-4)
Supplement: Supplementary file 3 — Supplementary Material 3. [file 42238_2024_220_MOESM3_ESM.pdf]

Table 3S. Observed Cannabinoids profile (%) of CBD, THCV, CBC, Δ^8-THC, CBG, and CBN in SD California.

Sample Code	CBD	THCV	CBC	Δ^8- THC	CBG	CBN
SD Cal 1	0.04	0.14	0.28	0.20	0.51	0.23
SD Cal 2	0.04	0.33	0.29	0.03	0.33	0.20
SD Cal 3	0.03	0.67	0.26	0.01	0.36	0.17
SD Cal 4	0.04	0.17	0.35	0.01	0.48	0.18
SD Cal 5	0.03	0.07	0.19	0.18	0.23	0.38
SD Cal 6	0.05	0.10	0.21	0.02	1.03	0.28
SD Cal 7	0.05	0.20	0.57	0.50	1.17	0.39
SD Cal 8	0.06	0.12	0.36	0.03	0.35	0.16
SD Cal 9	0.04	0.12	0.19	0.28	0.56	0.13
SD Cal 10	0.04	0.12	0.37	0.26	0.76	0.13
SD Cal 11	0.03	0.08	1.39	0.16	0.65	0.36
SD Cal 12	0.05	0.18	0.29	0.01	2.09	0.15
SD Cal 13	0.04	0.10	0.64	0.02	0.76	0.12
SD Cal 14	0.06	0.16	0.39	0.01	1.73	0.23
SD Cal 15	0.05	0.12	0.23	0.21	0.41	0.15
SD Cal 16	0.05	0.12	0.53	0.23	2.10	0.24
SD Cal 17	0.04	0.12	0.19	0.26	0.50	0.39
SD Cal 18	0.08	0.05	0.21	0.34	0.21	0.56
SD Cal 19	0.05	0.14	0.24	0.24	0.66	0.24
SD Cal 20	0.05	0.18	0.28	0.40	0.49	0.17
SD Cal 21	0.05	0.19	0.59	0.17	0.04	1.05
SD Cal 22	0.04	0.07	0.25	0.01	0.94	0.18
SD Cal 23	0.05	0.15	0.24	0.13	1.30	0.09
SD Cal 24	0.04	0.13	0.32	0.41	0.58	0.44
SD Cal 25	0.04	0.11	0.43	0.12	0.47	0.29
SD Cal 26	0.04	0.05	0.29	0.01	0.17	0.40
SD Cal 27	0.05	0.08	0.20	0.11	0.51	0.23
SD Cal 28	0.07	0.09	0.36	0.56	1.44	0.19
SD Cal 29	0.04	0.08	0.16	0.35	0.39	0.21
SD Cal 30	0.08	0.12	0.27	0.02	0.80	0.30
SD Cal 31	0.07	0.12	0.27	0.01	0.94	0.20
SD Cal 32	0.05	0.16	0.24	0.40	1.56	0.27
SD Cal 33	0.05	0.14	0.21	0.03	1.13	0.29
SD Cal 34	0.03	0.06	0.17	0.27	0.52	0.31
SD Cal 35	0.05	0.18	0.27	0.17	0.55	0.13
SD Cal 36	0.06	0.13	0.37	0.30	0.72	0.22
SD Cal 37	0.03	0.06	0.19	0.01	0.69	0.15

SD Cal 38	0.08	0.10	0.17	0.14	0.20	1.31
SD Cal 39	0.03	0.08	0.15	0.13	0.23	1.28
SD Cal 40	0.07	0.12	0.23	0.33	0.51	0.59
SD Cal 41	0.04	0.15	0.22	<0.01*	0.38	0.57
SD Cal 42	0.04	0.06	0.19	0.26	0.43	0.72
SD Cal 43	0.07	0.12	0.14	<0.01*	0.30	0.52
SD Cal 44	0.08	0.13	0.32	<0.01*	0.86	0.66
SD Cal 45	0.03	0.13	0.12	0.10	0.26	0.62
SD Cal 46	0.04	0.04	0.30	0.04	0.74	0.68
SD Cal 47	0.06	0.12	0.20	<0.01*	1.15	0.51
