# Supplementary material for: Evaluation of dispensaries’ cannabis flowers for accuracy of labeling of cannabinoids content
Source: J Cannabis Res. 2024 Mar 9;6:11. doi: 10.1186/s42238-024-00220-4 (PMC10924369; doi:10.1186/s42238-024-00220-4)
Supplement: Supplementary file 4 — Supplementary Material 4. [file 42238_2024_220_MOESM4_ESM.pdf]

Table 4S. Observed Cannabinoids profile (%) of CBD, THCV, CBC, Δ^8-THC, CBG, and CBN in CV California.

Sample Code	CBD	THCV	CBC	Δ^8- THC	CBG	CBN
CV Cal 1	0.05	0.11	0.24	0.17	1.08	0.29
CV Cal 2	0.05	0.07	0.20	0.22	0.62	0.37
CV Cal 3	0.08	0.13	0.25	<0.01*	1.05	0.22
CV Cal 4	0.04	0.06	0.18	0.35	0.23	0.33
CV Cal 5	0.07	0.09	0.33	<0.01*	0.38	0.44
CV Cal 6	0.04	0.57	0.28	<0.01*	0.33	0.13
CV Cal 7	0.06	0.06	0.24	0.17	0.60	0.24
CV Cal 8	0.02	0.09	0.24	0.26	0.43	0.60
CV Cal 9	0.07	0.17	0.54	0.47	0.75	2.47
CV Cal 10	0.08	0.17	0.47	0.23	1.13	0.19
CV Cal 11	0.07	0.10	0.27	<0.01*	0.18	0.23
CV Cal 12	<0.01*	0.09	0.31	0.27	0.84	0.16
CV Cal 13	0.04	0.08	0.19	0.08	0.61	0.47
CV Cal 14	0.04	0.10	0.28	<0.01*	0.63	0.26
CV Cal 15	0.06	0.10	0.27	0.44	1.13	0.31
CV Cal 16	0.05	0.08	0.22	0.40	0.75	0.28
CV Cal 17	0.04	0.12	0.70	0.30	0.75	0.46
CV Cal 18	0.05	0.76	0.28	<0.01*	0.35	0.34
CV Cal 19	0.05	0.14	0.31	0.00	1.68	0.23
CV Cal 20	0.11	0.08	0.03	0.43	0.64	0.40
CV Cal 21	0.18	0.11	0.24	0.17	1.08	0.29
